# Weighted enrichment method for prediction of transcription regulators from transcriptome and global chromatin immunoprecipitation data

**DOI:** 10.1093/nar/gkw355

**Published:** 2016-04-30

**Authors:** Eiryo Kawakami, Shinji Nakaoka, Tazro Ohta, Hiroaki Kitano

**Affiliations:** 1 Laboratory for disease systems modeling, RIKEN Center for Integrated Medical Sciences (IMS), Yokohama, Kanagawa 230-0045, Japan; 2 Department of Global Health Policy, Graduate School of Medicine, The University of Tokyo, Bunkyo-ku, Tokyo 113-0033, Japan; 3 Database Center for Life Science (DBCLS), Research Organization of Information and Systems (ROIS), Mishima, Shizuoka 411-8540, Japan; 4 The Systems Biology Institute, Minato-ku, Tokyo 108-0071, Japan; 5 Sony Computer Science Laboratories, Inc, Shinagawa-ku, Tokyo 141-0022, Japan; 6 Okinawa Institute of Science and Technology, Graduate University, Onna-son, Okinawa 904-0495, Japan

## Abstract

Predicting responsible transcription regulators on the basis of transcriptome data is one of the most promising computational approaches to understanding cellular processes and characteristics. Here, we present a novel method employing vast amounts of chromatin immunoprecipitation (ChIP) experimental data to address this issue. Global high-throughput ChIP data was collected to construct a comprehensive database, containing 8 578 738 binding interactions of 454 transcription regulators. To incorporate information about heterogeneous frequencies of transcription factor (TF)-binding events, we developed a flexible framework for gene set analysis employing the weighted *t*-test procedure, namely weighted parametric gene set analysis (wPGSA). Using transcriptome data as an input, wPGSA predicts the activities of transcription regulators responsible for observed gene expression. Validation of wPGSA with published transcriptome data, including that from over-expressed TFs, showed that the method can predict activities of various TFs, regardless of cell type and conditions, with results totally consistent with biological observations. We also applied wPGSA to other published transcriptome data and identified potential key regulators of cell reprogramming and influenza virus pathogenesis, generating compelling hypotheses regarding underlying regulatory mechanisms. This flexible framework will contribute to uncovering the dynamic and robust architectures of biological regulation, by incorporating high-throughput experimental data in the form of weights.

## INTRODUCTION

Most cellular processes and characteristics, such as metabolism, the cell cycle, differentiation and stress responses result from gene expression. Under the influence of environmental factors, cells change the expression of thousands of genes to synthesize appropriate amounts of functional proteins. The regulation of gene expression is mainly through transcription factors (TFs), which bind to specific DNA sequences and control the rate of RNA transcription. In addition, other proteins, including chromatin and DNA modifiers and co-regulators, influence the accessibility of binding sites by TFs. These combinations of transcription regulators provide elaborate control of gene expression and enable homeostatic reactions of cells in response to internal and external perturbations. Recently, it became clear that mature cells can be reprogrammed to achieve pluripotency by introducing only a few TFs (1). Thus, identifying responsible transcription regulators is an important first step in understanding regulatory mechanisms of both healthy and pathologic cellular states, as well as in manipulating cell differentiation.

Despite extensive research on TFs, it is still difficult to comprehensively ascertain the activation state of each TF because of the complicated layers of regulation involved in these systems. Multiple post-translational modifications, including phosphorylation, glycosylation, acetylation, ubiquitination and sumoylation, are necessary for TF function (2). Furthermore, TF binding to target sequences is affected by chromatin state and combinatorial interactions with other TFs and cofactors. Given that these states of TFs and chromatin are only measurable using antibodies, it is not practical to perform experiments to determine the TFs responsible for observed gene expression. On the other hand, we can now measure genome-wide mRNA expression levels using microarray or RNA-seq technology. Therefore, one of the most promising computational approaches is to predict which TFs are responsible for the mRNA expression using high-throughput transcriptome data.

To predict which TFs are responsible for a given set of transcriptome data, we first need to reconstruct relationships between TFs and their target genes, that is, a gene regulatory network (GRN). Recently, a method was proposed to model gene regulatory relationships based on predicted binding motifs of TFs (3). The authors constructed a linear model to explain expression of each gene in terms of predicted TF binding sites in proximal promoters. An alternative method uses chromatin immunoprecipitation (ChIP) data to link TFs to their target genes (4,5). By constructing sets of target genes for each TF based on ChIP data, the method can estimate transcription regulators likely responsible for observed gene expression patterns. Compared to motif-based methods, models based on ChIP data have the advantage of considering actual TF binding sites in a specific cellular state. At the same time, the use of such experimental data can also be a limitation of the method, since binding sites occupied by a given TF can differ markedly depending on the cell types or states in which they are measured.

Here, we present a novel approach for the prediction of TFs responsible for gene expression patterns, which incorporates data from vast numbers of mouse ChIP experiments, performed in various cell types and under a wide range of conditions. The method uses a weighted *t*-test in gene set analysis (GSA) to consider condition-dependent relationships between TFs and their target genes. Activities of mouse TFs predicted using this method were completely consistent with biological observations, regardless of cell type or conditions. Using case studies, we demonstrate the value of the method in capturing the dynamic and complex nature of gene regulation.

## MATERIALS AND METHODS

### ChIP-seq data analysis

Raw mouse ChIP-seq data files in SRA format were obtained from the GEO database and were converted to FASTQ format using the fastq-dump function of SRA Toolkit (http://www.ncbi.nlm.nih.gov/Traces/sra/sra.cgi?view=toolkit_doc&f=fastq-dump). Quality assessment of sequence reads was performed using FastQC (http://www.bioinformatics.babraham.ac.uk/projects/fastqc/). Each sequence read was aligned to the mouse mm9/NCBI37 genome using Bowtie2 version 2.2.5 with default parameters. To align sequences generated using the SOLiD sequencing system in colorspace, Bowtie version 1.1.2 was used with the colorspace indices built for mm9/NCBI37. Peaks were called using Model-based Analysis of ChIP-Seq (MACS) version 2.1 with default settings (*q* < 0.01).

### ChIP-array data analysis

Raw mouse ChIP-array data files were obtained from the GEO database. For Agilent and Nimblegen arrays, median-centering, quantile normalization and linear smoothing were performed using the Bioconductor packages Ringo and limma. Next, we identified ChIP-enriched regions using Ringo with estimated threshold *y*_0_ arising from the null distribution. For Affymetrix arrays, the Bioconductor package rMAT was used for normalization and smoothing. Then, enriched regions were identified with rMAT using a false discovery rate (FDR) threshold of 0.5.

### Annotation of ChIP peaks

Where original peak data (in narrowPeak, bed, wig or bigwig format) was available from the GEO database, peaks were converted into bed format. All binding peaks were converted to mm9 assembly by using the UCSC LiftOver Tool (6). The Bioconductor package ChIPpeakAnno was used to map the peaks and the regions surrounding transcription start sites (TSSs) (−5 to +2 kb) using TSS annotation data, ‘TSS.mouse.NCBIM37’.

### Nomenclature of TFs and target genes

Throughout this manuscript, we used Uniprot entry names (e.g. STAT3 and PO5F1) for representing TFs to distinguish them from the target gene name indicated by the Entrez gene symbol.

### Retrieving TF binding motifs

The Bioconductor package MotifDB was used to obtain DNA sequence motifs from JASPAR and UniPROBE databases. DNA sequences with >90% overlap of the motif were mapped to the proximal promoter region surrounding TSSs (−5 to +2 kb).

### Correlation analysis

Overlap of binding targets between different ChIP experiments of a TF was evaluated using Jaccard's coefficient. Spearman's rank correlation coefficient was employed for the evaluation of similarity in binding frequencies between pairs of TFs. Ward's method was employed for hierarchical clustering.

### Algorithmic details of wPGSA

We first evaluated the probability of a regulation based on the frequency of ChIP binding data. The probability of regulation was estimated by maximum-likelihood estimation. The likelihood function of a binomial distribution where *k* time bindings were observed in *n* ChIP experiments is denoted as:

\begin{equation*}L(p;k) = \frac{{n!}}{{k!(n - k)!}}{p^k}{(1 - p)^{n - k}};\end{equation*}

therefore, the binomial loglikelihood function is:

\begin{equation*}l(p;k) = K + k{\rm{log}}\ p + (n - k){\rm{log(}}1 - p{\rm{)}}\end{equation*}

where *K* is a constant that does not involve the parameter *p*. We can find the maximum of the likelihood function by setting the derivative of the loglikelihood function to zero and solving the equation for *p*:

\begin{equation*}\frac{{\partial l(p;k)}}{{\partial p}} = \frac{k}{p} - \frac{{n - k}}{{1 - p}} = 0\end{equation*}

\begin{equation*}\hat p = \frac{k}{n}\end{equation*}

Here, $\hat p$ denotes the maximum-likelihood estimate of the probability of regulation.

To evaluate whether a TF significantly changes the activity of a regulation, we compared the mean fold changes of gene expression of target genes of the TF against that of the background. We used weighted statistics for a two-sample *t*-test. Briefly, the weighted mean ${\bar E_T}$ of the fold changes *E* for a TF *T* was calculated using the formula:

\begin{equation*}{\bar E_T} = \frac{{\sum {{E_i}{p_i}} }}{{\sum {{p_i}} }}\end{equation*}

where *E_i_* is the fold change for gene *i* and *p_i_* is the estimated probability of the regulation of the gene by a TF. Fold changes for all genes were used without applying any threshold. The standard deviation of the weighted mean was calculated as:

\begin{equation*}{\sigma _T} = \sqrt {\frac{{\sum {{{({E_i} - {{\bar E}_T})}^2}} \sum {p_i}}}{{\sum {{p_i}} }}} \end{equation*}

Together with the mean ${\bar E_{all}}$ and the standard deviation ${\sigma _{all}}$ for all of the genes in the dataset, the weighted *t-*statistic for the TF is then:

\begin{equation*}{t_T} = \frac{{{{\bar E}_T} - {{\bar E}_{all}}}}{{\sqrt {\frac{{\sigma _T^2}}{n} + \frac{{\sigma _{all}^2}}{n}} }}\end{equation*}

where *n* is the total number of target genes. The degree of freedom (*df*) for this two-sample *t*-test with unequal variance is calculated as follows:

\begin{equation*}df = (n - 1)\frac{{{{\left( {\sigma _T^2 + \sigma _{all}^2} \right)}^2}}}{{\sigma _T^4 + \sigma _{all}^4}}\end{equation*}

Since the total number of target genes *n* is usually large enough, the *t*-statistic will have an approximately normal distribution and the *df* will have little effect on the *P*-value.

To correct the *P*-values for entire gene sets for multiple hypothesis testing, we calculated FDR q values using the Benjamini–Hochberg procedure (7).

The python code and the network between TFs and their targets for the wPGSA analysis used in this paper are available at https://github.com/eiryo-kawakami/wPGSA. The wPGSA web application and datasets are available at http://wpgsa.org. Users can use their own log fold change (LFC) data as an input to estimate the relative activities of TFs.

### Comparing enrichment methods

For the comparison, gsea2-2.2.2.jar was downloaded from the GSEA website (http://software.broadinstitute.org/gsea/) and GAGE was implemented in python based on the description of the authors (8).

### RNA-seq data analysis

RNA-seq data from myeloid cells stimulated with Lipopolysaccharide (LPS) and/or IL-10 (9) was obtained from the NCBI GEO database, using the GEO Series accession number GSE55385. Raw data files in SRA format were converted into FASTQ format using the fastq-dump function of SRA Toolkit. Each sequence read was aligned to the mouse mm9/NCBI37 genome using Tophat2 version 2.0.13 and Bowtie2 version 2.2.5 (10,11). Read counts for the genes annotated in UCSC mm9 were assembled using featureCounts (12), and differential gene expression was determined using DESeq2 version 1.8.1.

### Microarray data analysis

Microarray data were obtained from GEO Series accession numbers; GSE16375 and GSE31381 for the TF over-expression experiments in mESCs (13,14); GSE40728 for the experiments investigating CDK8-mediated STAT1 phosphorylation (15); GSE25044 for the IFN-α treated mouse embryonic fibroblasts (MEFs) (16); GSE10246 for the gene expression profiles across 91 murine cell and tissues types (17); GSE63786 for the time-course data from the lungs of influenza virus-infected mice (18). The Bioconductor package limma was used for median-centering, quantile normalization, linear smoothing and calculation of differential gene expression.

### Network construction of ES-specific TF interaction

We applied wPGSA to LFCs in embryonic stem cells (ESCs) compared with MEFs using the gene expression profiles obtained from GSE10246 datasets and calculated the Enrichment Score and FDR. Among 279 TFs with an FDR of <0.05, we further selected the ES-specific TFs that exhibited a marked Enrichment Score of >10 or ←10, and consequently obtained 37 TFs. The transcriptional regulatory network of TFs was constructed based on the frequency of ChIP binding of a TF to other TF genes. The network is a weighted directed network using the probabilities of TF binding as weights.

## RESULTS

### Reconstruction of GRNs by collecting global public ChIP data

To build a comprehensive picture of transcription regulation, we first collected all high-throughput experimental data evaluating mouse TF binding, including those from ChIP-seq and ChIP-chip experiments, from NCBI Gene Expression Omnibus database (GEO; www.ncbi.nlm.nih.gov/geo). Although an attempt was previously made to construct a similar mammalian ChIP database (ChEA2; http://amp.pharm.mssm.edu/ChEA2/index.html) (19), the recent dramatic decrease in the cost of high-throughput assays has led to a rapid increase in ChIP experiments, covering hundreds of TFs. We integrated additional data obtained from the GEO database with the ChEA2 database to construct a ChIP database containing a total of 8 578 738 binding interactions for 454 transcription regulators from 87 ChIP-chip and 530 ChIP-seq publications comprising 273 ChIP-chip and 2924 ChIP-seq individual experiments (Supplementary Table S1). This database is four times larger than the ChEA2 database, although it currently contains only mouse ChIP data. The data also includes ChIP-seq data from the mouse ENCODE project (20) as the ChEA2 database. The detailed TF binding peak data, including the locations of the peaks and the distances to the nearest TSSs, are available at http://wpgsa.org/download/.

**Figure 1.**
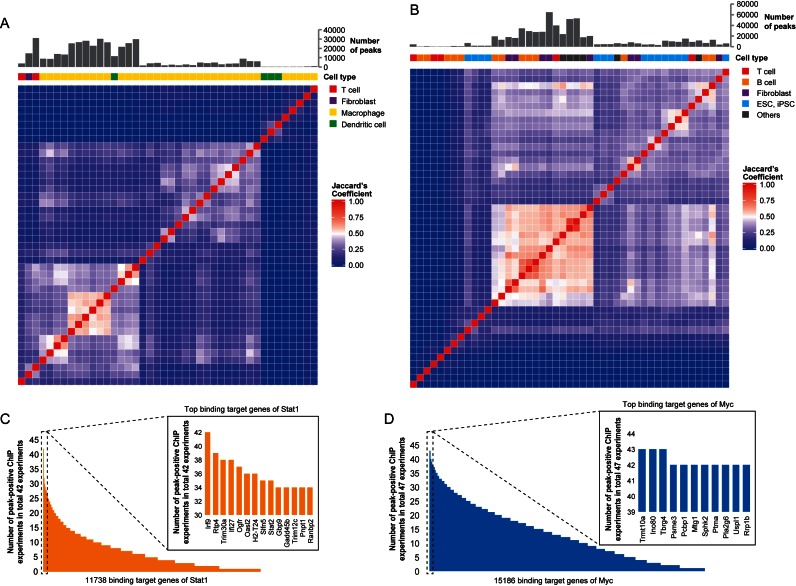
Overlap of binding targets between different ChIP experiments. Hierarchically clustered similarity heatmaps of binding targets between pairs of individual ChIP experiments investigating (**A**) STAT1 and (**B**) MYC. Jaccard's coefficient was used to evaluate the degree of overlap between individual experiments. Over the heatmap, the number of peaks detected in the experiment is indicated as a bar chart and the cell types used in the experiment are shown in color boxes. Binding frequencies for potential target genes observed in various ChIP experiments investigating the TFs (**C**) STAT1 and (**D**) MYC. Target genes with a high frequency of TF binding are presented in additional panels at high magnification.

To evaluate the overlap of binding targets between different ChIP experiments, we calculated the Jaccard's similarity between each pair of individual ChIP experiments for a TF and hierarchically clustered them (Figure 1A and B). Unexpectedly, we observed rather large differences between experiments, even where both the same antibody and the same cell type were used. For both STAT1 and MYC TFs, the majority of experiments demonstrated <50% overlap in shared binding targets. This likely reflects the complicated condition dependency of TF binding, which involves not only cell type, but also culture conditions and environmental stimuli. The multistep procedures of ChIP experiments, in each of which, optimization of conditions is required, might also lead to inconsistency between the experiments undertaken by different labs. Although we found limited overlaps between different experiments, there were a few genes bound by a TF throughout most of the experiments (Figure 1C and D). Thus, we found rather large differences in binding frequency of a TF between target genes, suggesting that some genes are controlled by the TF regardless of conditions, while others are more conditional.

**Figure 2.**
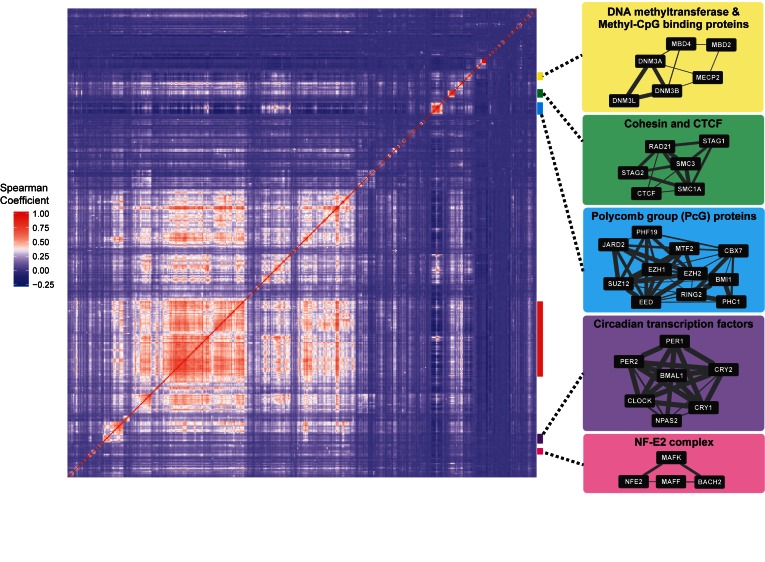
A hierarchically clustered similarity heatmap of binding targets between pairs of TFs. Spearman's rank correlation coefficient was used to evaluate the similarities in binding between the TF pairs. Some characteristic clusters corresponding to known protein families, or complexes with cooperative regulatory functions, are indicated at the right of the heatmap. The protein–protein interaction (PPI) network of each cluster was obtained from the STRING database v10 (http://string-db.org) (26). The width of the line represents the experimental score of the STRING database. A giant cluster containing TFs sharing many binding targets with one another and also with transcription suppressors, such as PcG proteins, is indicated in red.

Next, we examined the similarity in binding targets between each pair of TFs to unravel cooperative relationships between TFs. We identified some characteristic clusters in the heat map (Figure 2 and Supplementary Table S2). One of them was the Polycomb-group (PcG), a family of proteins that modify histones and/or mediate interactions of distant regulatory elements to silence mostly target genes by forming Polycomb repressive complexes (21,22). In addition to the dense interactions within the cluster, PcG proteins also shared binding targets with many other TFs, indicating their wide range of counteracting roles in transcriptional suppression. Other characteristic clusters corresponded to known protein families or complexes with cooperative regulatory functions, including Methyl-CpG binding proteins, cohesion complex proteins and TFs related to the control of circadian rhythm (23–25). Notably, we found dense protein–protein interactions (PPIs) between TFs within those clusters (Figure 2). In addition, the ratio of high confidence PPI in the STRING database (26) (experimental score > 0.5) was significantly higher between TFs with high similarities in binding (Spearman's rank correlation coefficient > 0.5) than between TF pairs with low similarities (odds ratio = 7.23, *P* = 3.31 × 10^−101^; Fisher's exact test, Supplementary Table S3). These results indicate that similarities in binding targets partially reflect physical interactions between TFs. A giant cluster containing many TFs was found in the lower left of the heat map (Figure 2, red line). Components of the cluster shared many binding targets with each other, and also with transcription suppressors such as PcG proteins. This suggests an underlying architecture of transcription regulation where some genes are redundantly controlled by various TFs.

Finally, we evaluated the correlation between ChIP binding sites and the sites for the corresponding DNA sequence motif obtained from JASPAR and UniPROBE databases (27,28). For ∼60% of motifs, we found a significantly higher frequency of ChIP binding peaks for the TF in gene promoters containing at least one copy of the motif sequence (Supplementary Table S4; *P* < 0.05, Mann–Whitney U-test), indicating that the motifs do represent ChIP binding sites. Interestingly, TFs demonstrating inconsistency between ChIP binding sites and locations of the corresponding motif had been investigated in a significantly smaller number of ChIP experiments (*P* = 4.54 × 10^−6^; Welch's *t*-test). This indicates that prediction of TF targets or motifs based on such small numbers of ChIP experiments can place a disproportionate emphasis on a limited number of experimental conditions. In addition, the correlation between frequencies of ChIP binding and numbers of motif sites around a TSS was low (Supplementary Table S4; Pearson correlation coefficient = −0.003 to 0.36). Thus, although the TF binding events are basically defined by promoter sequences, the frequencies of binding events are strongly influenced by other factors.

Overall, our integrated ChIP database provides a comprehensive picture of transcription regulation, including condition dependency of TF binding and cooperative relationships between TFs. Although we observed inconsistencies between individual ChIP experiments, combining the data from many observations masked technical variations, and revealed the control exerted by transcription regulators.

### Overview of the weighted parametric gene set analysis (wPGSA) method

GSA has been widely used for the detection of biologically relevant gene categories (29,30). Recently, GSA methods employing the *t*-test were proposed to compare a parametric distribution of gene sets against a background distribution of all genes (8,31–32). Parametric GSA methods have advantages in terms of sensitivity and computational effort, compared with other GSA methods based on Hypergeometric or rank tests (31). To utilize information regarding heterogeneous frequencies of TF binding events, we introduced a weighted *t*-test procedure (33) into a parametric GSA for the prediction of TFs responsible for a specific gene expression pattern.

**Figure 3.**
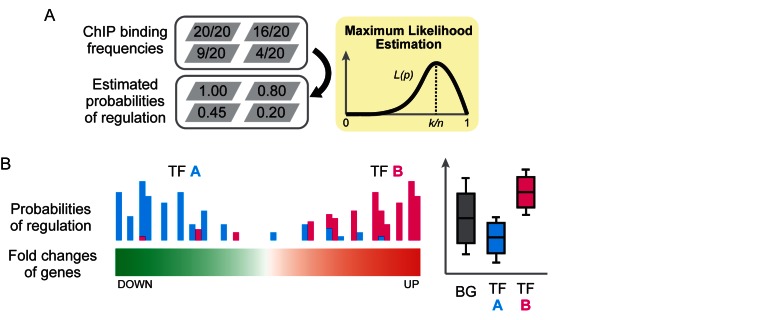
A schematic overview of the wPGSA algorithm. (**A**) Evaluation of the probability of regulation based on the frequency of TF ChIP binding. When *k* times bindings are observed in *n* ChIP experiments, the likelihood function of probability of regulation has a maximum value at *k*/*n*. (**B**) Weighted two-sample *t*-tests using the probabilities of regulation as weights. In the weighted *t*-test procedure, weighted statistics were used to place more emphasis on samples with high frequencies of TF binding. For example, TF A binds more frequently to those genes with low expression, while TF B has frequent binding peaks on highly expressed genes. In such cases, the weighted mean of the fold changes is low for TF A and high for TF B.

The wPGSA for TF prediction comprises three steps:

**Step 1: evaluation of the probability of regulation**. We evaluate the probability of regulation based on the frequency of ChIP binding of a TF. The probability of a TF regulating a gene, where *k* times binding events were observed in *n* ChIP experiments, can be readily estimated as *k*/*n* using the maximum-likelihood method (Figure 3A).

**Step 2: calculation of an Enrichment Score based on a weighted *t*-test**. We tested whether the mean LFCs in expression of target genes of a TF are significantly different from that of the whole genes Since the traditional fold change, calculated simply as a ratio of expression values, is sometimes inappropriate for the evaluation of differential expression, especially in the case of low expression genes, we recommend the use of the ‘moderated’ fold change calculated by limma (34) or DESeq2 (35). To put more emphasis on those genes with high frequencies of TF binding, we employ a weighted two-sample *t*-test, using the probabilities of regulation as weights. For the weighted *t*-test, we use the weighted mean and variance statistics to calculate a weighted *t-*statistic (Figure 3B). As the *t*-statistic denotes how many standard errors the weighted mean of the LFCs in expression of the selected gene set are away from that of the background, we use the weighted *t*-statistic as an enrichment score indicating the relative activity of a TF.

**Step 3: determining the significance level of Enrichment Scores, taking into account multiple hypothesis testing**. The weighted *t*-statistic follows the *t*-distribution as described in the ‘Materials and Methods’ section. We first calculated a *P*-value for the null hypothesis that the weighted mean LFCs in expression of target genes of the TF are equal to that of the background, using the weighted *t*-statistic and the *t*-distribution. To correct the *P*-values computed for individual TFs for multiple testing issue, the FDR is calculated using the Benjamini–Hochberg procedure (7). The FDR is the estimated probability that the Enrichment Score is falsely judged as significant. In this study, TFs with FDRs of <0.05 are considered to significantly change their activities.

The detailed implementation is described in the Methods section. The wPGSA will be applicable for the prediction of other responsible factors where incorporating the strength of the relationship as a weighting factor is desirable.

### Validation of the wPGSA method for TF prediction

**Figure 4.**
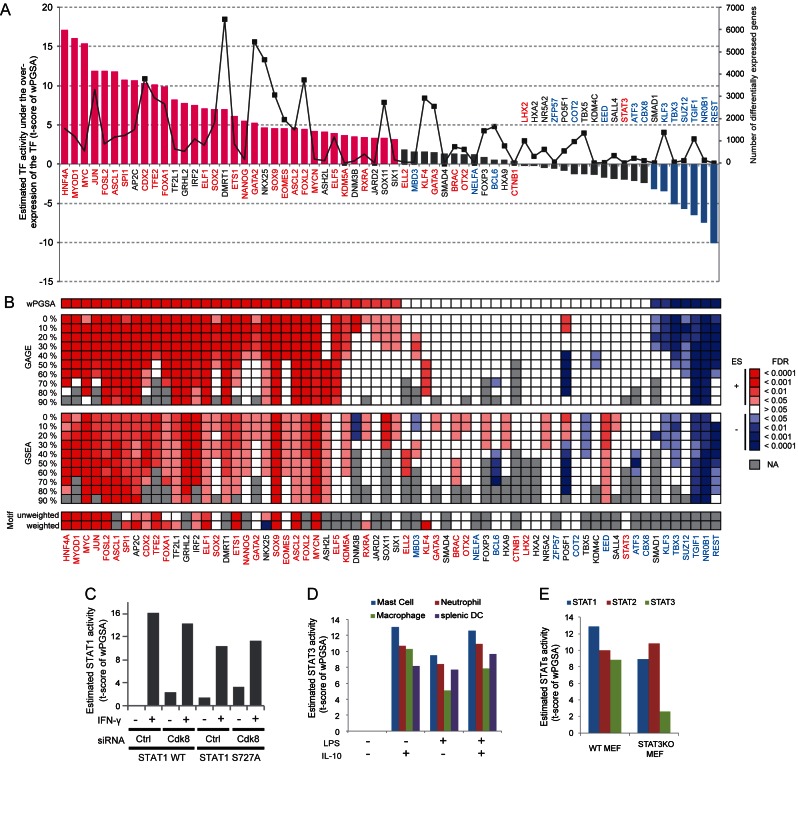
Validation of wPGSA predictions incorporating ChIP data. (**A**) Enrichment Scores of 65 TFs under conditions of their own over-expression. Magenta bars indicate significantly active, and blue bars significantly repressive, effects. TFs with names in red text are known as transcription activators, TFs with names in blue are primarily transcription repressors and TFs in gray characters have either dual (activator and repressor) or uncharacterized roles. The gray line indicates numbers of differentially expressed genes under conditions of TF over-expression. We considered gene expression changes of >1.5-fold and FDR < 0.05 as significant. (**B**) Comparison of TF regulations identified as significant in the TF-over-expression experiment by various enrichment procedures. Colored boxes indicate the FDR levels, in which deeper red colors indicate a smaller FDR with a positive Enrichment Score and deeper blue colors indicate a smaller FDR with a negative Enrichment Score. (**C**) STAT1 activities of wild-type or S727A MEFs estimated by wPGSA. MEFs treated with the siRNAs, siCdk8 or siCtrl, were either stimulated with IFN-γ or unstimulated. (**D**) STAT3 activities of mouse myeloid cells treated with IL-10 and/or LPS. (**E**) Activities of STAT family proteins of WT or STAT3 KO MEFs stimulated with IFN-α.

To validate the TF activities predicted by wPGSA using biological observations, we first applied the method to transcriptome data from mouse embryonic stem cells (mESCs) over-expressing various TFs (13,14). Among the 65 TFs evaluated, 34 were estimated to be significantly activating in data from samples where they were over-expressed, while 7 exhibited significant negative Enrichment Scores, indicating repressive effects on their target genes (Figure 4A and Supplementary Table S5). The majority of TFs with significant positive Enrichment Scores are known transcription activators. We also confirmed that the 6 of 7 TFs with significantly negative Enrichment Scores mainly act as transcription repressors. Thus, the activities of over-expressed TFs predicted by wPGSA were in excellent agreement with those expected from their known biological roles. Three TFs, NELFA, MBD3 and STAT3, which exhibited Enrichment Scores opposite to those expected were reported to have relatively little effect on the transcriptome in the source studies (13,14), indicating insufficient over-expression (Figure 4A, gray line). Prediction based on such subtle gene expression changes can lead to inappropriate results affected by background noise.

We then compared the performance of wPGSA with those of other enrichment methods based on unweighted statistical tests. GSEA with the gene labels permutation option (30), which is based on the rank test, and GAGE (8), a parametric enrichment method based on two sample *t*-tests, were applied to TF over-expression datasets as control methods. To this end, the weighted gene sets used in wPGSA were converted into conventional unweighted gene sets by setting discrete thresholds of 0–90%. For instance, when we set a 30% threshold, those genes with a 30% or more frequency of ChIP binding of a TF were considered as regulatory targets of the TF. We used these ChIP-based gene sets instead of the C3 TF motif gene set in MSigDB (http://software.broadinstitute.org/gsea/msigdb/collections.jsp) to compare the methods in the same condition as much as possible. The TF regulations estimated by the three methods overlapped substantially, but the predictions made by GSEA and GAGE were affected by the thresholds for the gene sets (Figure 4B). With a low threshold, too many genes with low frequencies of TF binding in a gene set might present obstacles to obtaining an accurate prediction, while too high a threshold could lead to inappropriate predictions by excluding important target genes. Although the sensitivity of wPGSA was globally higher than that of GSEA and GAGE, we observed some TFs, such as KLF4, BRAC, BCL6 and ATF3, for which the predictions by GSEA and/or GAGE were more consistent with their known biological roles. This indicates that sometimes it might be better to ignore completely the low frequency, possibly non-specific, bindings of a TF to predict its regulation.

Next, we compared TF prediction based on ChIP binding data to DNA sequence motif-based prediction. Sites for a motif around a TSS, rather than ChIP binding sites, were used to associate TFs with genes. When the motif-based enrichment method was applied to the transcriptome data from over-expressed TFs, we obtained smaller absolute Enrichment Scores for both activators and repressors, to those based on ChIP data (Figure 4B and Supplementary Table S6). Moreover, some TFs, such as GATA2 and EOMES, which were predicted by ChIP-based wPGSA to have significantly altered activities under conditions of over-expression, gave insignificant results using the motif-based method. Even when we used the number of DNA sequence motif sites around a TSS as a weight for wPGSA, the Enrichment Scores scarcely improved (Figure 4B and Supplementary Table S6). Overall, wPGSA incorporating ChIP binding data as weights more clearly estimated the relative activities of TFs than the motif-based method.

Some TFs are constitutively expressed and only activated by phosphorylation in response to specific stimuli. We examined whether our wPGSA method can predict such post-translational activation of TFs. To this end, microarray data from MEFs treated with interferon-γ (IFN-γ), which induces phosphorylation of STAT1 at Y701 and S727 leading to increased transcriptional activity, was used (15). As shown in Figure 4C, wPGSA predicted STAT1 activity to be significantly increased under conditions of IFN-γ stimulation. Silencing of CDK8, which is reported to reduce S727 phosphorylation of STAT1 without affecting Y701 phosphorylation, partially reduced the predicted STAT1 activity. In addition, the S727A mutant of STAT1 exhibited a lower Enrichment Score using wPGSA than that under CDK8 silencing, reflecting complete absence of S727 phosphorylation. The fact that STAT1 activity remained even in the S727A mutant indicates that STAT1 activity is determined by both Y701 and S727 phosphorylation. Thus, wPGSA elaborately predicted phosphorylation-mediated STAT1 activities, depending on several regulatory conditions. For validation of estimations of STAT3 activity, we applied the method to RNA-seq data from myeloid cells stimulated with LPS and/or IL-10 (9). LPS stimulation induces MAPK-driven Y705 phosphorylation of STAT3, whereas IL-10 induces canonical S727 phosphorylation (15,36–37). We found greatly elevated STAT3 activity in all myeloid cells under IL-10 stimulation (Figure 4D). Simultaneous treatment with both LPS and IL-10 showed comparable levels of STAT3 activation, while relatively lower but significant Enrichment Scores were observed under LPS stimulation alone. These results suggest that canonical S727 phosphorylation and non-canonical Y705 phosphorylation work independently and are not cumulative.

We also checked whether our method could distinguish between TFs with highly similar binding motifs, such as those of the STAT family. In wild-type MEFs treated with interferon-α (IFN-α) (16), STAT1, STAT2 and STAT3 were all predicted to be highly active (Figure 4E). STAT3 knockout drastically decreased only the STAT3 Enrichment Score, while the activity of other STATs remained relatively unaffected. The expression level and phosphorylation status of STAT1 and STAT2 are reported to remain comparable when STAT3 expression is knocked down (16), suggesting that our method could clearly discriminate the activities of the different STAT TFs, despite their highly similar binding motifs.

In summary, these results demonstrate that wPGSA based on ChIP data can predict activities of various TFs, regardless of cell types and conditions, with results that are completely consistent with biological observations.

### Creating comprehensive pictures of TF activity using ChIP-based wPGSA

**Figure 5.**
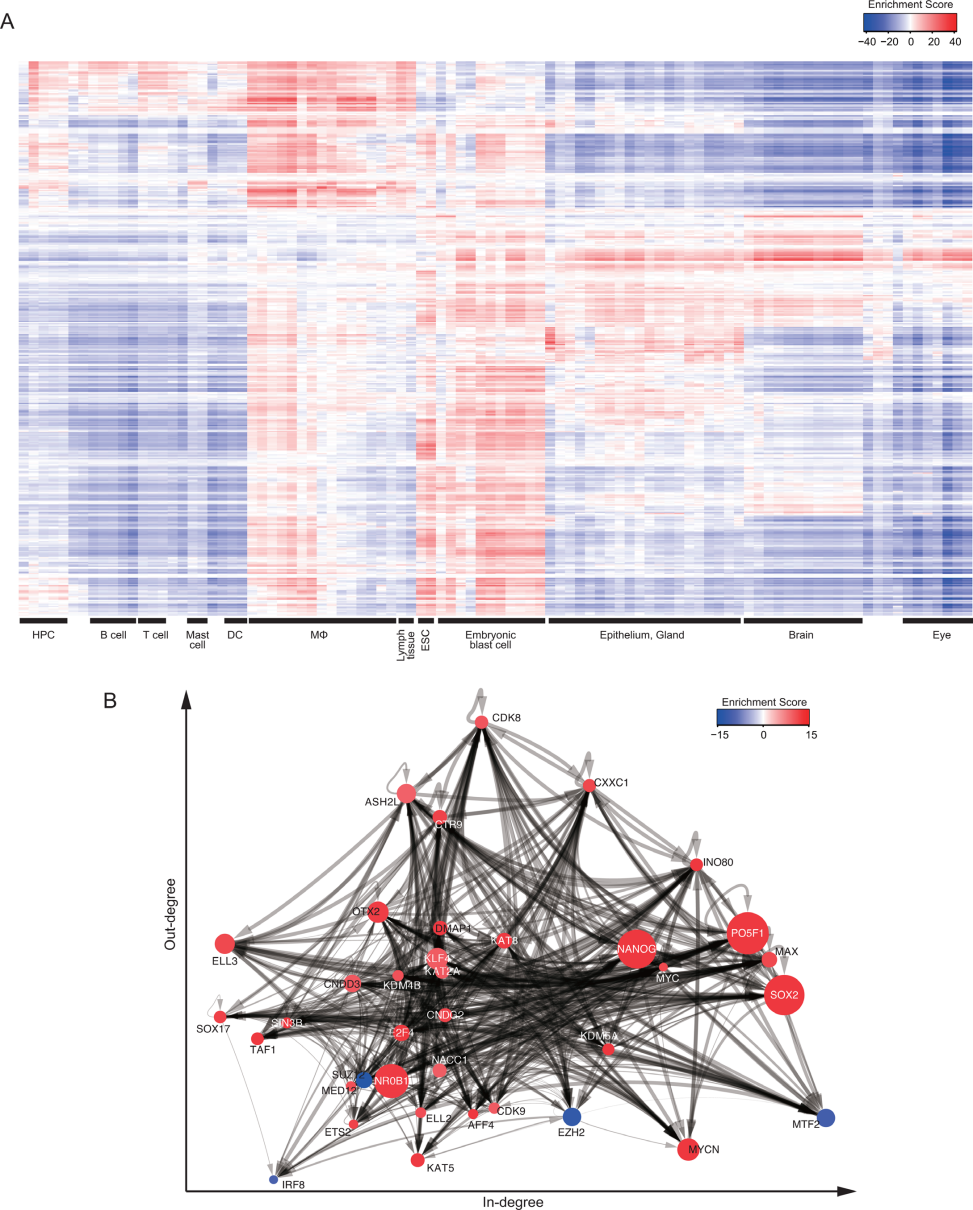
Activities of TFs characterizing cell types and tissues. (**A**) Relative TF activities across 91 murine cell types and tissues estimated using wPGSA. The vertical axis of the histogram denotes 486 TFs and the horizontal axis 91 cell types and tissues. Enrichment Scores for both vertical and horizontal axes were hierarchically clustered using Ward's method. The Pearson's correlation coefficient was used to evaluate similarities. Characteristic groups of cell types and tissues are labeled under the horizontal axis. HPC: hematopoietic stem cell; DC: dendritic cell; MΦ: macrophage; ESC: embryonic stem cell. (**B**) Transcriptional regulatory network of TFs specific for mESCs (Enrichment Score of >10 or ←10). Those nodes with more out-degrees (regulate many other TFs) were placed in a higher position and those with high in-degrees (regulated by many other TFs) were placed to the right. The node sizes indicate the LFCs of the TF gene in mESC compared with MEFs as the input and the node colors indicate the Enrichment Scores of TFs estimated with wPGSA using the LFCs of all genes. The width of the directed line represents the probability of transcriptional regulation of a TF over other TF gene.

To illustrate the utility of wPGSA for capturing the dynamic and complex nature of gene regulation, we applied the method to gene expression profiles from 91 murine cell types and tissues (17). LFCs, compared to the median expression across all cell types, were used to estimate the relative activities of TFs in each cell type. Our results revealed activations and repressions of TFs that characterize cell types and tissues (Figure 5A and Supplementary Table S7). For instance, inflammatory TFs, including IRF2, SPIB and IRF8, were activated in myeloid cells, while they were repressed in other tissues. In addition, a characteristic activation of BRDT was observed in testis, which is consistent with the central role of this TF in the regulation of spermatogenesis (38). Interestingly, we also found high similarity between the TF activation patterns of ESCs and those of embryonic blast cells, including MEFs. This result explains why MEFs can be reprogrammed into induced pluripotent stem cells (iPSCs) through forced expression of a few TFs (1). The four TFs required for reprogramming of MEFs, PO5F1, SOX2, KLF4 and MYC, showed high levels of activation in ESCs and targeted extensively those TFs highly activated or repressed specifically in ESCs (Enrichment Score of >10 or ←10) and were also extensively targeted by those TFs (Figure 5B). Thus, these four factors might interact closely with other ES-specific TFs to maintain pluripotency.

**Figure 6.**
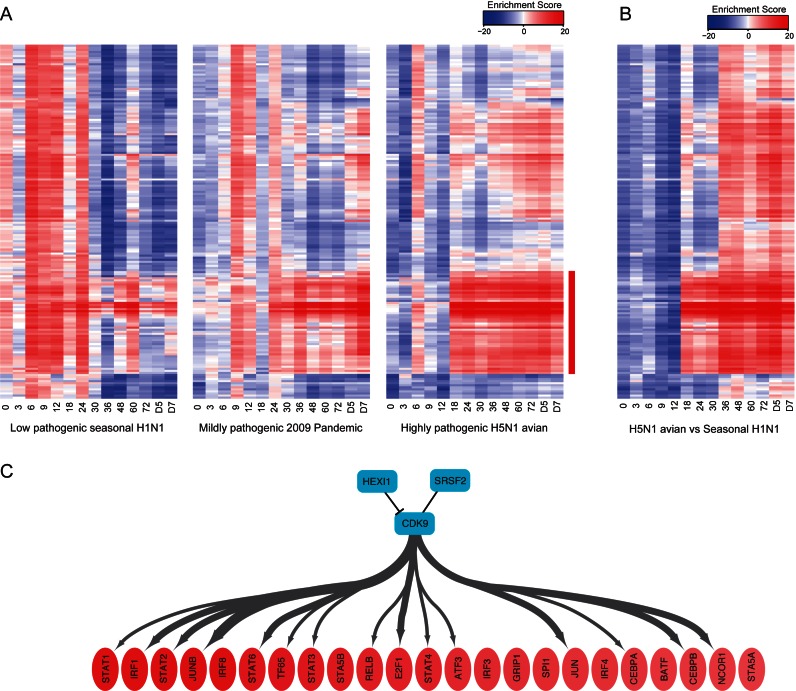
Temporal changes in predicted TF activities during influenza virus infection. (**A**) Enrichment Scores were calculated using wPGSA based on gene expression changes of influenza virus infected mouse lungs compared to lungs mock-infected with PBS. Highly activated or repressed TFs with absolute values of Enrichment Scores of >20 for at least one time point are shown. (**B**) Temporal changes in the Enrichment Scores calculated with wPGSA using gene expression changes of mouse lungs infected with the highly pathogenic H5N1 influenza virus compared to seasonal H1N1 virus. TFs are represented on the vertical axis in the same order as (A). (**C**) Transcription regulation relationship between CDK9 and TFs highly activated at the late phase of H5N1 virus infection. Width of the directed line represents the probability of transcriptional regulation for each TF. Inhibitory or cooperative physical interactions between P-TEFb components are represented as black lines.

Next, we applied the method to compare the time-course transcriptome data of mouse lungs infected with influenza viruses with those of mock-infected lungs, including the low pathogenicity seasonal H1N1 virus, the mildly pathogenic virus from the 2009 pandemic season, and the highly pathogenic H5N1 avian virus (18). Although these influenza A viruses exhibit significant differences in virus titers and inflammatory responses during the infection, the gene regulatory mechanisms that account for the different profiles have not been elucidated. We observed activation of various TFs at the early phase of seasonal H1N1 infection, whereas these TFs were less active early in the 2009 pandemic and H5N1 infections (Figure 6A and Supplementary Table S8a–c). By contrast, inflammatory TFs, including the IRF and STAT families were highly activated 18 h post-infection especially with the H5N1 virus (Figure 6A, indicated in red). These temporal changes in TF activities did not appear to correlate with lung virus titers, since H5N1 virus produced the highest lung titers throughout the infection (18). This indicates a potential mechanism of the pathogenic virus that grows silently, inhibiting early immune responses and causing late acute inflammation. When we compared the H5N1 virus to the seasonal H1N1 virus, the TF CDK9 exhibited high repressive effects from 0 to 12 h post-infection, coincident with overall repression of inflammatory TFs (Figure 6B and Supplementary Table S8d). CDK9 is a catalytic subunit of the positive transcriptional elongation factor b (P-TEFb), which consists of CDK9 and cyclin T, and RNA polymerase II (39). Moreover, CDK9 targeted the majority of TFs predicted to be highly activated (average Enrichment Score of >10) in the late phase of infection (after 8 h post infection) (Figure 6C). Taken together with a previous report that P-TEFb interacts with influenza A virus polymerase (40), we hypothesize that pathogenic influenza viruses strongly inhibit CDK9-dependent activation of inflammatory TFs to escape from the early immune response.

Thus, wPGSA incorporating ChIP data clearly captured comprehensive pictures of gene regulation that characteristically and dynamically change among cell types and during disease progression. Moreover, we identified some potential key regulators of cell reprogramming and influenza virus pathogenesis, generating compelling hypotheses about underlying regulatory mechanisms.

## DISCUSSION

A major goal of molecular biology is to identify the key regulators and elucidate regulatory mechanisms of gene expression responsible for healthy and pathologic cellular states. Although high-throughput technologies now allow us to comprehensively measure genome-wide changes in mRNA expression, uncovering underlying regulatory mechanisms is an ongoing challenge due to their complex and condition-dependent nature.

In this study, we developed a novel framework for GSA based on the weighted *t*-test, namely wPGSA, which can incorporate vast ChIP datasets in the form of weights. Compared with motif-based estimation, wPGSA based on ChIP data has several advantages. First, it can consider the strength of a relationship as a weight. Although TF-binding motifs determined by SELEX or ChIP experiments represent consensus sequences to which TFs are likely to bind, information relating to the frequency of actual TF binding is not included in the motif. By putting more emphasis on those genes with high frequencies of actual TF binding, wPGSA can predict which TFs are responsible for a gene expression pattern with more sensitivity and reliability. Second, we can assess the activities of a larger number of TFs with wPGSA based on ChIP data, because limited numbers of TF-binding sites have been converted into motifs; only 202 non-redundant motifs for all vertebrates TFs were contained in the JASPAR CORE database in 2014 (27). Moreover, using the motif-based method, TFs with similar binding motifs, such as the STAT family, are barely distinguishable, despite the fact that each TF can be activated independently under different conditions. Our method can assess the activity of any TFs for which high-throughput ChIP experiments have been performed, and distinguish them from one another. Therefore, while binding motifs are useful in investigation of the DNA-binding properties of TFs, for the prediction of TF activity, it is more efficient and effective to use direct ChIP binding data.

Finally, we would like to discuss the limitations and the extensibility of wPGSA. In this study, we considered only proximal TF binding sites, although most mammalian genes are regulated by a complex array of enhancers. TF binding to proximal sites dominantly contributes to gene regulation (41), which is one of the reasons why our method could reasonably predict the regulation for most of the TFs evaluated in this study. By contrast, promoter-distal enhancers have an important role in the control of cell-type specificity (42). Recently a genome-wide atlas of enhancers was constructed covering the majority of tissues and cell types of human and mouse (43,44). We are currently developing methodology for the evaluation of enhancer-mediated gene regulation by integrating high-throughput experimental data capturing genome conformation such as Hi-C into the enhancer atlas. In addition, other biological regulatory mechanisms, such as post-transcriptional regulation by microRNAs and metabolic control by enzymes, are also highly condition-dependent and vary in strength. To obtain a holistic view of biological systems, we should integrate multiple types of omics data corresponding to different layers of the biological network (45). wPGSA will also be applicable to the integration of different types of high-throughput experimental data in the form of weights. The wPGSA framework will help uncover the dynamic and robust architectures of biological regulatory systems responsible for cellular states in health and disease.

## Supplementary Material

Supplementary Data

SUPPLEMENTARY DATA
