# Supplementary material for: Weighted enrichment method for prediction of transcription regulators from transcriptome and global chromatin immunoprecipitation data
Source: Nucleic Acids Res. 2016 Apr 30;44(11):5010–21. doi: 10.1093/nar/gkw355 (PMC4914117; doi:10.1093/nar/gkw355)
Supplement: SUPPLEMENTARY DATA [file supp_44_11_5010__index.html]

Weighted enrichment method for prediction of transcription regulators from transcriptome and global chromatin immunoprecipitation data — SUPPLEMENTARY DATA 

# Weighted enrichment method for prediction of transcription regulators from transcriptome and global chromatin immunoprecipitation data

## SUPPLEMENTARY DATA

- SUPPLEMENTARY DATA
- SUPPLEMENTARY DATA
- SUPPLEMENTARY DATA
- SUPPLEMENTARY DATA
- SUPPLEMENTARY DATA
- SUPPLEMENTARY DATA
- SUPPLEMENTARY DATA
- SUPPLEMENTARY DATA
